# Public health information needs in districts

**DOI:** 10.1186/1472-6963-13-S2-S12

**Published:** 2013-05-31

**Authors:** Ties Boerma

**Affiliations:** 1 Department of Health Statistics and Information Systems, World Health Organization, Geneva, Switzerland

## 

Following the decentralization of health services in line with Primary Health Care declaration in Alma Ata in 1978, districts have gradually become a cornerstone of health systems in sub-Saharan Africa. Though administrative systems vary, the majority of the over 4,000 districts in the region have a population between 200,000 and 400,000 people, and most have an administrative structure that relies on district health management teams.

Health information is a critical component of district health systems, and is essential for district health teams to effectively plan and manage health services. The five projects described in this special issue all centrally feature health information systems, address different aspects of district health information systems in innovative ways, and will generate important evidence on a relatively neglected area of health systems [1].

What are the essential public health information requirements of districts?

First, districts need to generate and report data for the national level on a range of indicators. District reports provide regular data on health service provision, morbidity and mortality (including immediate reporting of notifiable diseases), but are also the basis for national and subnational data on infrastructure, health workforce, financing, etc. To be consistent, comparable and reliable, reporting systems need to adhere to national and international standards.

Second, district public health information systems need to generate relevant information for the local planning, management and monitoring of services. Key parameters include population distribution; health facilities and workforce; budget and expenditures by programme and facilities; access to and quality of services; coverage of interventions; and epidemiological information. To meet these requirements, a continuous process of data generation, compilation, analysis, dissemination and use for resource allocation is essential [2,3]. For information to be useful for decision making at the district level, districts should have sufficient control over the allocation of financial and other resources, including personnel.

Districts usually do not select their own indicators for monitoring progress and performance. Most countries have selected between 20 and 40 core indicators with baselines and targets to monitor their national health sector strategic five-year plans. A full national health plan may have many additional indicators that serve to monitor the implementation of the plan’s different aspects. In addition, specific health and disease programmes have their own indicators and targets. The rationalization of data collection and reporting by health facilities and districts has to be part of efforts to improve information systems [4].

The bulk of health information is generated through a small number of data sources: facility recording and reporting, registration of vital events, household surveys, facility assessments, and administrative databases. Each of those data sources can provide key information for districts.

The main vehicle for reporting health data, and for regular monitoring, is the national health management information system (HMIS) based on health facility reports. The problems of such systems are well known. Health workers often have a heavy data collection and reporting burden; much of the gathered data are not used; and incomplete and inaccurate reporting affect data quality. For a dozen or so indicators, such as immunization, institutional delivery and outpatient utilization, it is possible to estimate district coverage rates by making assumptions about denominators (e.g. the expected number of deliveries in the district). These estimates provide relevant information to district managers and allow comparisons across districts, and in some countries, such as Uganda, district league tables are produced to rank the performance of districts using an index that includes a range of coverage indicators obtained from the health facility reporting system. It is good to keep in mind, however, that there is considerable uncertainty in the denominators – the estimated target population – as these may deviate considerably from the actual population because they are based on census projections and because service utilization is pragmatic and not confined to district boundaries. As a result, district coverage estimates from facility data often have great uncertainty. Provincial or regional estimates are often more reliable, and have the advantage of being comparable to survey-based coverage estimates. A fairly recent HMIS development is the use of the internet to expedite reporting from districts (or health facilities) to the national level, such as the District Health Information System (DHIS) which is now used by more than 30 countries. Such developments increase the potential for greater investment and use of data at the district level.

Information on mortality and causes of death is often lacking even nationally, as reliable death registration systems are not in place [5]. The national census, generally conducted every ten years, can be a source of district specific child mortality estimates in some countries. Hospital data can provide a general idea of cause of death patterns, but are biased and tend to be unreliable as standardized disease classification procedures – namely the International Classification of Diseases (ICD-10) – is often not used. A notable exception is the recent work in Mozambique where an electronic reporting system, with a thorough revision of coding and certification practices, greatly improved the quality of cause of death data from hospitals [6]. Many districts, however, lack reliable information, and in this case, data on causes of death and burden of disease profiles are often generated from a small number of longitudinal community demographic surveillance sites that collect data on probable cause of death through verbal autopsy (6). In Tanzania, resulting disease profiles were incorporated in a planning and budget tool that is used by all districts to guide resource allocation.

Household surveys are a critical data source for monitoring progress and performance at the national level. Only a few national surveys have an adequate sample size to allow district level estimates for key indicators such as immunization coverage or skilled birth attendance (e.g. Malawi Demographic and Health Survey in 2010). District surveys are also conducted as part of research, but are too costly, both in terms of technical and financial resource requirements, to be conducted on a large scale. Even the relatively simple immunization coverage cluster sample surveys have never reached scale in district applications. Several innovations hold the promise of making conducting household surveys easier (including automated household sample selection, electronic data entry and compilation, as well as analysis and report production), but surveys remain a resource-intensive exercise.

Basic data on the distribution of the population, health facilities (public and private) and health workforce are critical for district health managers. These data allow computation of key management indicators (such as workload), and administrative data through medicines and logistics management systems provide continuous information for performance monitoring and management of the system. Services readiness is another critical element, which can be assessed in supervisory visits using checklists, or more systematically in a facility assessment using a standardized tool such as the Service Availability and Readiness Assessment (SARA) that includes the availability of trained staff, basic equipment, diagnostics and medicines (currently at the national level, but could be adapted for district level needs) [8].

A major weakness in many district public health information systems is the lack of capacity to analyze and synthesize data from the multiple systems to inform decision making. Most districts have a health information officer as part of the district management team, and may have additional capacity in disease programmes, but the ability to assess data quality and assemble different indicators is often limited. Information technology provides a major opportunity to facilitate this process, but in general analytical capacity strengthening requires much more attention, which is addressed by one of the studies in this volume [1]. Unfortunately, the envisioned monitoring and evaluation support function of the next administrative level – region or province – has not materialized for the health sector in many countries. National level support is often limited simply because there are too many districts in proportion to the national support capacity in the Ministry of Health or Bureau of Statistics. Innovative approaches to support district capacity have to be found.

In summary, districts need a health information system that draws from multiple data sources. Reliable mortality and cause of death information primarily relies on hospital data and mortality profiles generated by community studies. Provincial or regional estimates from household surveys can provide an indication of child mortality levels and trends and of coverage of major interventions. The main continuous sources of information for districts are, however, locally generated health facility and administrative data. Ultimately, the use of information for decision making at the district level requires that districts actually have control over allocation of financial and other resources, such as staff. Much can be done to improve the district health information systems, especially if the introduction of information technology is done in combination with a review and rationalization of data collection, to the benefit of local health services as well as national monitoring and evaluation systems.

## Competing interests

The author serves as an Expert Advisor to the Doris Duke Charitable Foundation’s African Health Initiative.

## Declarations

This article has been published as part of *BMC Health Services Research* Volume 13 Supplement 2, 2013: Improving primary health care to achieve population impact: the African Health Initiative. The full contents of the supplement are available online at http://www.biomedcentral.com/bmchealthservres/supplements/13/S2. Publication of this supplement was supported by the African Health Initiative of the Doris Duke Charitable Foundation.
